# Where Women Access Contraception in 36 Low- and Middle-Income Countries and Why It Matters

**DOI:** 10.9745/GHSP-D-21-00525

**Published:** 2022-06-29

**Authors:** Sarah E. K. Bradley, Tess Shiras

**Affiliations:** a Abt Associates, Rockville, MD, USA.

## Abstract

We examined where women access modern contraceptives, using recent Demographic and Health Survey data from 36 low- and middle-income countries and disaggregating results by contraceptive method, age, marital status, residence, socioeconomic status, and country income. We used bivariate and multivariate regression analysis to assess how demographic factors are associated with contraceptive source. In pooled analysis across countries analyzed, we found that 34% of users rely on private sources, 63% use public sources, and 3% use other sources. Among private sector users, 41% use pharmacies or drug shops, 11% general shops or markets, 36% private hospitals and clinics, and 12% nongovernmental or faith-based organizations. This analysis demonstrates the importance of the private sector for specific population segments including women in the wealthiest population quintile (odds ratio [OR]: 4.09, *P*<.001 compared with women in the poorest quintile), adolescents (OR: 2.03, *P*<.001 compared with women ages 40–49), never married women (OR 1.55, *P*<.001 compared with ever-married women), and urban women (OR: 1.42, *P*<.001 compared with rural women). The private sector does not only serve these populations, however. On average across countries analyzed, 22% of the poorest contraceptive users and 27% of rural users use private sources. Leveraging both sectors is key to meeting the reproductive needs of women across all sociodemographic groups in low- and middle-income countries.

## Abstract (precis)

The public and private sectors are both important sources of modern contraception in nearly every low- and middle-income country studied and across sociodemographic groups. Catalyzing cross-sectoral collaboration and leveraging the potential of both sectors are critical as countries work to expand access to modern contraception and meet women's reproductive intentions.

## Teaser

**Key Findings:**

- Both the public and the private sectors are key sources of modern contraception, and the private sector has maintained a relatively stable share of the contraceptive market.
- Our analysis shows a clear pattern between source and modern contraceptive method. Although the private sector is primarily a source for short-acting resupply methods (e.g., condoms and contraceptive pills), our analysis demonstrates that some injectable, implant, and IUD users rely on private sources for these methods.
- The private sector is disproportionately used by unmarried women, adolescents, urban women, and wealthier women. However, the private sector also serves 1 in every 4 of the poorest contraceptive users and more than 1 of every 4 rural users, on average across countries.

**Key Implications:**

- Understanding where women access modern contraception is an important first step for policy makers and program implementers to enhance contraceptive access and equity.
- Harnessing the potential of all market actors—government, nongovernmental, and private commercial—is key to accelerating progress toward countries' family planning goals to expand contraceptive access and choice and meet the reproductive needs and preferences of current and potential future contraceptive users.

## BACKGROUND

Across the 69 poorest countries in the world, an estimated 314 million women are using modern contraception.^1^ While much attention and many resources have focused on galvanizing voluntary contraceptive demand, counting additional users, and improving availability of specific methods, less attention has been devoted to understanding where women obtain their contraceptives and how source patterns differ by contraceptive method and by women's sociodemographic characteristics. Frequently, efforts to reach family planning goals focus primarily on government resources and the public sector, ignoring the important role that the private sector (including private clinics, pharmacies, drug shops, shops, and nongovernmental organizations [NGOs]) can and does play. Examining where women obtain their contraception is key to informing collaboration across sectors and ultimately expanding contraceptive access and choice and meeting women's contraceptive needs.

Understanding where women access contraception is key as more governments and donors engage with the private sector to support national reproductive health goals. For example, family planning costed implementation plans often include concrete plans to collaborate with the private sector. Many countries' costed implementation plans also focus on adolescents, who disproportionately rely on the private sector to access family planning methods. In addition, donors such as the Bill & Melinda Gates Foundation, the United States Agency for International Development (USAID), and the World Health Organization have prioritized the need to systematically engage the private sector to meet health goals.^2^^–^^5^

Previous studies investigating sources of modern contraception in low- and middle-income countries (LMICs) are largely limited to data from 2013 and earlier or are limited to a small sample of countries.^6^^–^^11^ The most recent multicountry analysis of sources for contraception in LMICs used Demographic and Health Survey (DHS) data from 2010 to 2015 from 33 sub-Saharan African countries to compare source patterns between younger and older modern contraceptive users, and it highlighted the need to improve contraceptive access, choice, and quality for adolescents, in particular, by focusing efforts on sources used disproportionately by youth.^12^

We build on previous analyses by examining contraceptive source patterns in recent DHS data from 36 LMICs spanning 3 regions. This study provides the most updated nationally representative estimates of contraceptive sources, addressing 3 primary research questions:

1. Currently, where do women obtain their contraceptives, and how do sources vary by region, country, and contraceptive method?
2. Within the private sector, what are the relative roles of private clinics, pharmacies and drug shops, general shops and markets, and NGOs in providing contraception?
3. How do source patterns vary by women's age, marital status, geography, socioeconomic status, and country income?

This study provides the most updated nationally representative estimates of contraceptive sources.

Within each of these questions, we explore the close relationship between contraceptive source and method mix.

## METHODS

### Study Setting and Data

To answer the primary research questions, we analyzed data from women aged 15–49 years surveyed in 36 LMICs. We used the most recent DHS data from every FP2020 focus country that conducted a survey since 2013: Afghanistan 2015, Bangladesh 2014, Benin 2017–2018, Burundi 2016–2017, Cambodia 2014, Chad 2014–2015, Democratic Republic of the Congo (DRC) 2013–2014, Egypt 2014, Ethiopia 2016, Gambia 2013, Ghana 2014, Guinea 2018, Haiti 2016–2017, India 2015–2016, Indonesia 2017, Kenya 2014, Lesotho 2014, Liberia 2013, Malawi 2015–2016, Mali 2018, Myanmar 2015–2016, Nepal 2016, Nigeria 2018, Pakistan 2017–2018, Philippines 2017, Rwanda 2014–2015, Senegal 2017, Sierra Leone 2013, Tajikistan 2017, Tanzania 2015–2016, Timor-Leste 2016, Togo 2013–2014, Uganda 2016, Yemen 2013, Zambia 2013–2014, and Zimbabwe 2015.

DHS asks nationally representative samples of women about their contraceptive use and where they obtained their current method. Because our analysis is focused on sources for contraception, we included only women who were currently using a method of contraception for which the source was routinely asked about in DHS: women who reported currently using condoms, pills, injectables, intrauterine devices (IUDs), implants, or male or female sterilization, and those we classified as using an “other modern method” (diaphragms, contraceptive foam/jelly, female condoms, and emergency contraception). Women using one of these other modern methods were included in the analysis, but they were not examined separately by method owing to small sample sizes. While male and female sterilization are 2 distinct modern contraceptive methods, we combined them into one method for this analysis because male sterilization has a small sample size and because the sources used for male and female sterilization are the same. We excluded women using the lactational amenorrhea method, standard days, and other fertility awareness methods, and any method coded in a DHS dataset as “other modern,” as surveys did not systematically ask for sources of these methods. Women who did not report a source for their contraception were excluded from analysis.

In 5 countries (Afghanistan, Bangladesh, Egypt, Pakistan, and Yemen), the DHS did not collect family planning information from never-married women, so results for these countries reflect ever-married women only. We excluded data from these countries when results were disaggregated by marital status. Unless otherwise noted, results from all other countries are presented for all women, not just married women, to accurately portray contraceptive sources among all users, married and unmarried.

We weighted all analyses using DHS sampling weights. In the pooled analyses described below, we combined data from all interviewed women in each of the selected surveys and multiplied the sampling weight by a country-specific constant defined as:

$$\frac{\Sigma_{1}^{n}w_{i}}{nw_{i}},$$

where *w_i_* is the weighted number of interviewed women in survey *i*, and *n* = 1, 2, … 36 surveys included in analysis.

This constant equalized the effective weighted sample size across countries, so each country contributed equally to the analysis and results are not weighted more heavily toward surveys with larger sample sizes. We considered weighting results by the population size of each country but found that nearly three-fourths of the population-weighted sample would be from Asia because the Asian countries in our analysis are so populous. We repeated this analysis with population weights, and the results were not substantively different, although averages looked more similar to results from Asia. Pooled results presented here should be interpreted as averages across countries analyzed. Similarly, regional results are not representative of the entire region but should be interpreted as the average across countries analyzed in each region.

In regression analysis, we excluded the small proportion of contraceptive users who obtained their method from friends, family members, and “other” sources (Table 1) to examine predictors of obtaining modern contraception from private sector versus public sector sources. We used these adjusted pooled sampling weights in regression analyses. The regression model included country fixed effects and accounted for the complex design of each survey sample.

**TABLE 1.** Classifications for Family Planning Sources

Private clinical	Private hospitals, clinics, doctors, nurses, midwives, health centers, maternity homes, and other private medical
Private pharmacy or drug shop	Pharmacy, drug shop, dispensary, and chemist
Private shop or market	Shop, market, bar, disco, vending machine, gas station, grocery store, guest house/hotel, warehouse, and other private
NGO/FBO	Mission hospital, mission health center/clinic, church, mosque, religious institution, NGO health facility, NGO mobile clinics, and NGO CHW
Public	All public sources including hospitals, clinics, and CHWs
Other	Friend, relative, partner, parent, traditional healer, traditional birth attendant, school, the respondent, and other

Abbreviations: CHW, community health worker; FBO, faith-based organization; NGO, nongovernmental organization.

### Measures

The key measure analyzed was the source where women last obtained their current contraceptive method. Types of contraceptive sources reported varied across countries, so we worked with country experts to standardize the classification of each source into the 5 categories shown in Table 1, largely following the source categories used in previous analyses (e.g., Campbell et al.^6^). Private sector sources are more heterogeneous than those in the public sector.

The key measure analyzed was the source where women last obtained their current contraceptive method.

We examined source patterns by socioeconomic status by using the DHS wealth quintiles, which divide the population surveyed in each country into evenly sized quintiles based on their household assets. We used the bottom and top quintiles, respectively, to represent women from the poorest 20% and wealthiest 20% of households in each country. Additionally, we analyzed source patterns by country income, using gross national income (GNI) per capita (purchasing power parity adjusted).

## RESULTS

### Current Contraceptive Sources

On average across countries analyzed, more than 1 in 3 women who use modern contraception obtains her method from a private sector source (34%). Sixty-three percent use public sources, and 3% use other sources such as a partner, friend, or relative (Table 2).

**TABLE 2.** Sources of Contraception Among Modern Contraceptive Users, by Region, Method, and Select Demographic Characteristics^a^

Category	Private, %	Public, %	Other, %	Total, %	No.
Geography					
All 36 countries	63	34	3	100	380,244
Asia	41	56	2	100	287,646
East and Southern Africa	26	71	3	100	52,415
West and Central Africa	31	65	4	100	21,910
Method					
Condom	61	28	11	100	44,322
Pill	52	47	1	100	56,189
Injectable	26	73	1	100	55,731
Implant	13	86	1	100	18,850
IUD	29	70	1	100	23,987
Sterilization	24	74	3	100	180,311
Marital status					
Married	30	68	2	100	338,010
Unmarried	47	43	9	100	10,450
Residence					
Urban	45	51	3	100	130,214
Rural	27	70	2	100	250,030
Socioeconomic status					
Poorest 20%	22	76	2	100	61,544
Wealthiest 20%	50	46	4	100	83,892

Abbreviation: IUD, intrauterine device.

a Pooled data from 36 Demographic and Health Surveys.

Women go to different sources for different contraceptive methods, as shown in Table 2. On average across analyzed countries, the majority of women using short-acting resupply methods, specifically condoms and pills, obtain them from private sector sources (61% and 52%, respectively). For injectables, long-acting reversible contraceptives (LARCs), and permanent methods (PMs), the public sector is the primary provider. However, approximately one-fourth of women who use IUDs, injectables, or sterilization go to private sector sources for their method (29% for IUDs, 26% injectables, and 24% sterilization). In addition to the public and private sectors, other sources—primarily friends, husbands, or other family members—provide condoms to 11% of users of that method.

Women also use different sources for contraception in different regions of the world. On average across the Asian countries analyzed, 41% of users obtain their method from private sector sources (Table 2). In the West and Central African countries analyzed, an average of 31% of users rely on private sources, and on average across East and Southern African countries analyzed, 26% go to a private source.

**FIGURE 1:**
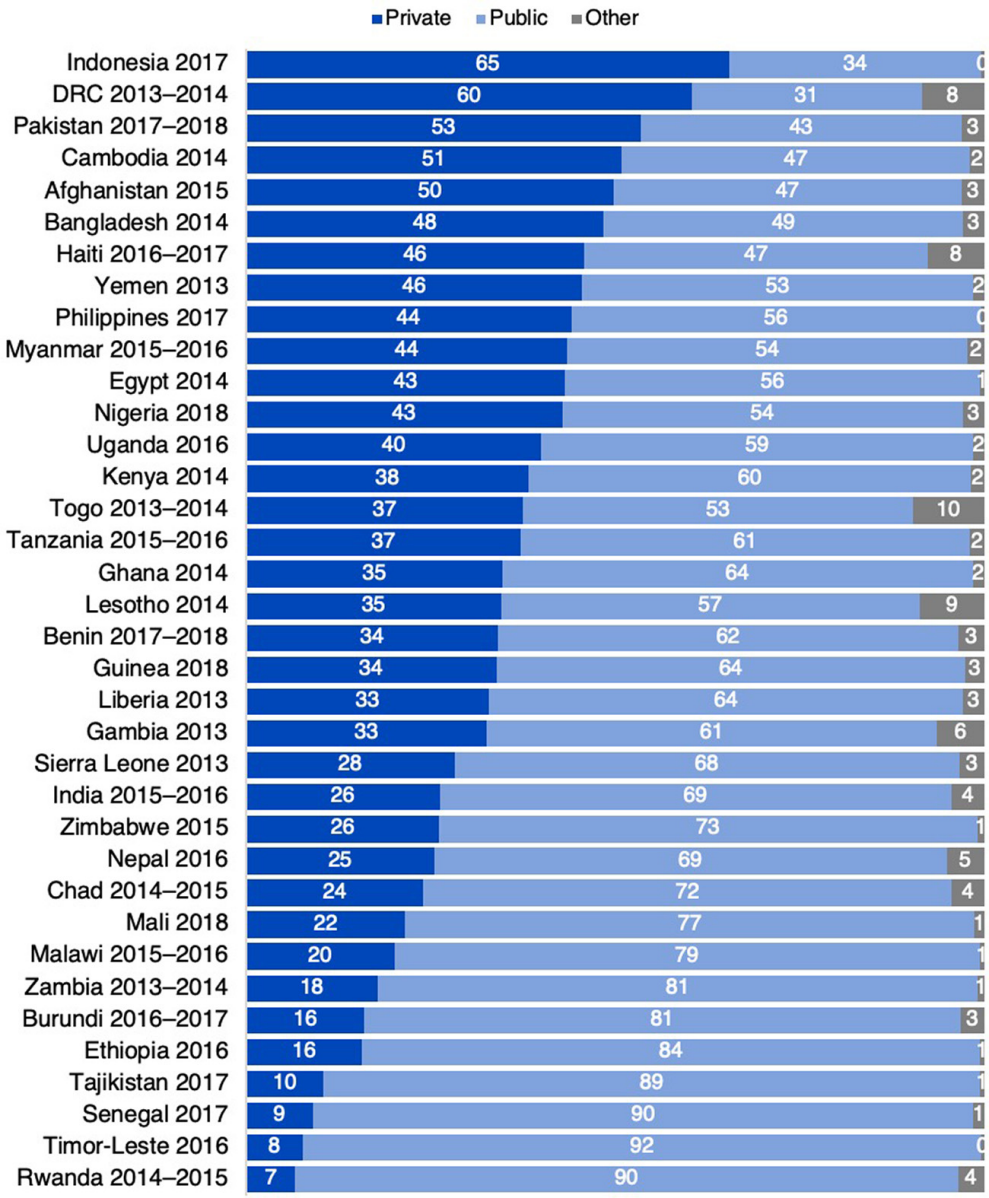
Sources of Contraception Among Modern Contraceptive Users, by Country Abbreviation: DRC, Democratic Republic of the Congo.

Regional averages hide some of the more dramatic variations in contraceptive sources at the country level (Figure 1). More than half of women using modern contraception go to private sector sources in Indonesia, DRC, Pakistan, Cambodia, and Afghanistan. In contrast, more than 80% of family planning users in Zambia, Burundi, Ethiopia, Senegal, Tajikistan, Timor-Leste, and Rwanda obtain their method from a public source.

### Contraceptive Sources Within the Private Sector

**FIGURE 2:**
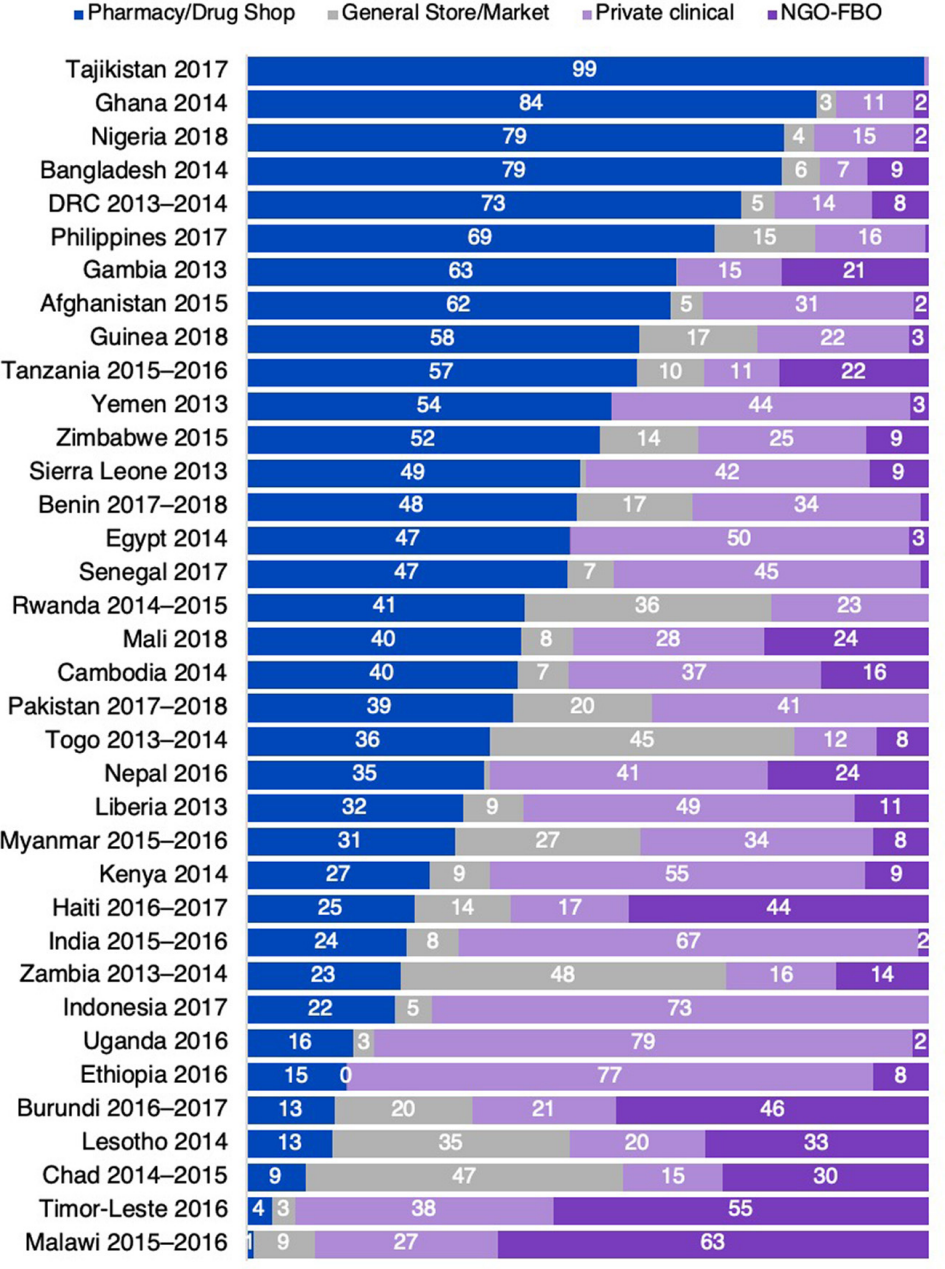
Private Sector Sources of Contraception, by Country Abbreviation: FBO, faith-based organization; NGO, nongovernmental organization; DRC, Democratic Republic of the Congo.

Narrowing our focus to women who go to private sector sources for contraception, we find that on average across the 36 countries studied, 41% of women obtain their method from pharmacies or drug shops and 11% from general shops or markets (Table 3). Therefore, more than half of private sector users obtain contraception from nonclinical sources (pharmacies or shops). Hospitals and clinics are an important private sector source, too: on average across countries, more than 1 in 3 (36%) private sector users go to these sources. NGOs and faith-based organizations (FBOs) have a more limited role on average (12%) but play a much larger role in certain countries, as shown in Figure 2.

**TABLE 3.** Private Sector Sources of Contraception Averaged Across All Countries, by Region, by Method^a^

	Category	Private Clinics, %	Pharmacies and Drug Shops, %	NGOs and FBOs, %	Shops and Markets, %	Total, %	No. of Private Sector Contraceptive Users
Geography	All 36 countries	36	41	12	11	100	109,020
	Asia	38	45	6	10	100	80,744
	East and Southern Africa	36	26	21	17	100	13,295
	West and Central Africa	25	54	8	12	100	6,686
Method	Condom	5	56	4	34	100	23,492
	Pill	12	72	5	11	100	32,203
	Injectable	62	19	18	1	100	16,372
	Implant	62	2	35	1	100	2,661
	IUD	88	1	11	<1	100	7,678
	Sterilization	76	<1	24	0	100	26,209

Abbreviations: FBO, faith-based organization; IUD, intrauterine device; NGO, nongovernmental organization.

a Pooled data from 36 Demographic and Health Surveys.

Among women who go to private sector sources for contraception, more than half use nonclinical sources.

The private sector comprises different types of sources in different regions (Table 3). The private sector is primarily nonclinical in West and Central African countries analyzed, where an average of 66% of private sector users obtain their method from pharmacies or drug shops, shops, or markets (Box 1). Private hospitals and clinics play a larger role, on average, in Asian countries analyzed (38%) and East and Southern African countries analyzed (36%).^9^ NGOs and FBOs are most important in East and Southern Africa, serving more than 1 in 5 (21%) of private sector users.

BOX 1 **Expanding Contraceptive Access Through Drug Outlets in Tanzania**

Accredited drug dispensing outlets (ADDOs) in Tanzania have received substantial investments from the government and donors in recent years, and their use has increased from 33% to 59% among private sector family planning clients. Tanzania's modern contraceptive prevalence rate has also increased in that time from 20% to 27%. Continued support to ADDOs can further expand contraceptive access and choice, particularly if policies are designed to allow and programs are implemented to train ADDOs to supply a range of methods. Read more at https://www.shopsplusproject.org/sources-family-planning-materials.

Great diversity exists across the private sector landscape in each country (Figure 2). More than 75% of private sector users in Tajikistan, Ghana, Bangladesh, and Nigeria obtain their contraception from pharmacies or drug shops. In contrast, private clinics and hospitals serve the majority of private sector users in Uganda, Ethiopia, Indonesia, India, and Kenya. While the role of NGOs and FBOs is limited in most countries, they are the dominant private sector source in Malawi and Timor-Leste and serve more than 40% of private sector contraceptive users in Burundi and Haiti.

Private sector sources are different for different methods. Pharmacies and drug shops are, on average across countries analyzed, the dominant private source for pills (72%) and condoms (56%). Shops and markets are also a key source for private sector condom users (34%). In East and Southern African countries analyzed, shops and markets play a larger role, selling condoms to 60% of private sector condom users (data not shown). Nearly 1 in 5 (19%) private sector injectable users obtain their method from a pharmacy or drug shop, on average, which aligns with an increasing number of national policies that allow pharmacies or drug shops to sell injectables. Private clinics, however, are the primary private sector source for injectables and for LARCs and PMs. NGOs and FBOs play the largest role (35%) for private sector implant users. While the very small 1% of users who report obtaining their injectable from a shop or market or their implant from a pharmacy or drug shop may seem surprising, these women likely purchased their method from 1 source and had it administered or inserted by a different provider. Overall, the private sector source mix in each country is closely related to the method mix. For example, private clinics are the dominant private sector source (73%) in Indonesia and injectables are accordingly the most used method in the country.

### Variation in Contraceptive Sources

#### Age

On average across the 36 countries, private sector use is highest among the youngest users (ages 15–19, 41%; date not shown) and lowest among the oldest age group (40–49, 31%; data not shown), with a steady pattern of decreasing private sector reliance as women age. This source pattern aligns very closely with method mix patterns by age. On average across the 36 countries, younger women primarily rely on short-acting methods, while older women are much more likely to use LARCS and PMs. For example, as expected, sterilization—a method for which nearly three-quarters of users rely on public sources—is much more common among users aged 40–49 years (29%, not shown) compared with no detectable use among 15- to 19-year-olds (0%; data not shown) and 1% among users aged 20–24 years. Conversely, condoms—the method most commonly sought from private sources—are much more common among the youngest than oldest users (29 versus 8%; data not shown). Notably, use of pills (a short-acting method) and implants (a LARC) are used at similar levels across age groups.

Private sector use is highest among the youngest users and lowest among the oldest age group, with steadily decreasing private sector reliance as women age.

**FIGURE 3:**
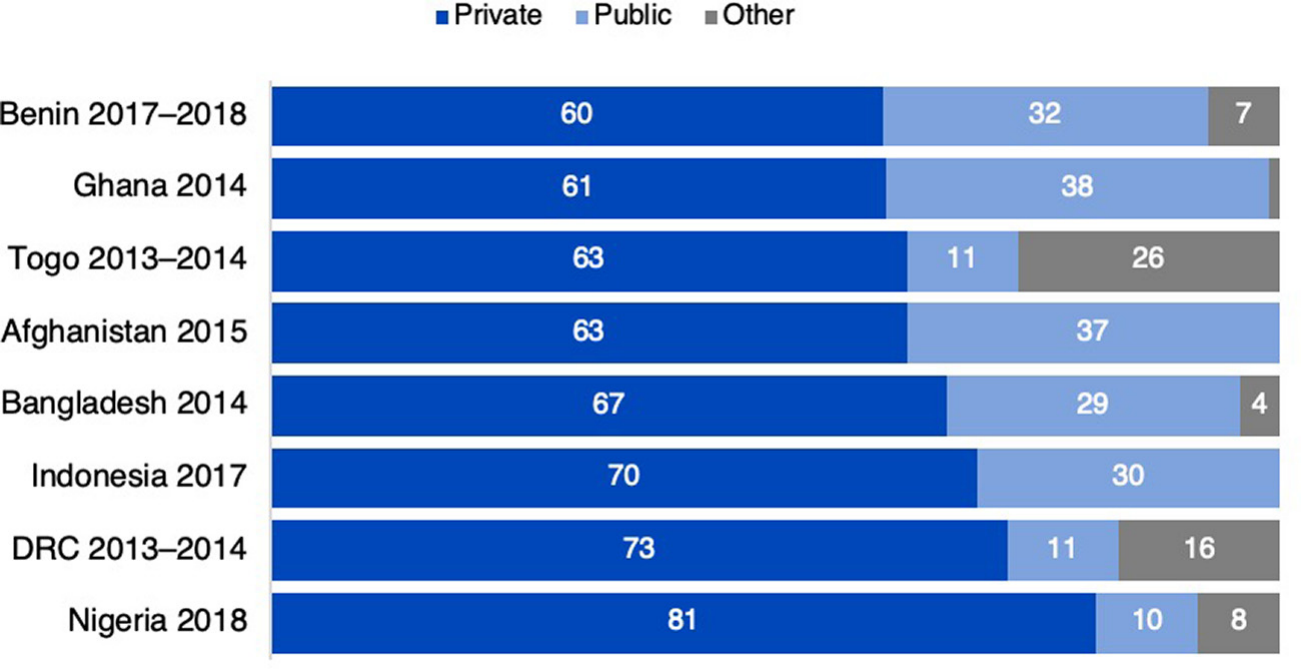
Sources for Contraception Among Adolescent Users (Ages 15–19 Years) in Selected Countries Abbreviation: DRC, Democratic Republic of the Congo.

Because many countries have stated goals to improve their family planning services reaching adolescents,^13^ we include Figure 3 to highlight where adolescent users obtain contraception in a few countries that have sufficient sample sizes. More than 80% of adolescent users in Nigeria rely on private sector sources for their contraception, along with 73% and 70% in the DRC and Indonesia, respectively. In Bangladesh and Afghanistan, where only married women were interviewed, more than 60% of (married) adolescent users obtain contraception from private sources. Private sector use among adolescents is also at or above 60% in Togo, Ghana, and Benin. In many of these countries, “other” informal sources—mostly partners, friends, and family members—supply a substantial portion of contraceptives. In DRC and Togo, adolescents use these informal sources at higher rates than the public sector.

#### Marital Status

Source and method mix patterns by marital status echo patterns by age, as older women are more likely to be married. On average across the 32 countries analyzed by marital status, unmarried users are far more likely than married users to obtain their method from private sector sources (47% versus 30%, Table 2) and to use short-acting methods (84% versus 66%). The largest difference in method mix between unmarried and married contraceptive users is the level of condom use, which is nearly 8 times higher among unmarried users (45% versus 8%; data not shown).

#### Urban and Rural Residence

On average across countries analyzed, 45% of urban contraceptive users access their method from private sector sources (Table 2). While private sector use is less common in rural areas, more than 1 in every 4 rural contraceptive users (27%) obtains her method from a private source on average across the 36 countries analyzed. In many countries, method mix also differs by place of residence. However, on average across analyzed countries, the method mix is largely consistent in urban and in rural areas. The 2 methods that differ are condoms, which are more common in urban areas (17% versus 9%; data not shown), and injectables, which are more common in rural areas (37% versus 27%; data not shown).

On average, 45% of urban contraceptive users access their method from private sector sources, while private sector use is less common in rural areas.

On average, 93% of women in the poorest wealth quintile in their country also live in rural areas. Likewise, 83% of women in the wealthiest quintile in their country live in urban areas. Therefore, the findings on contraceptive sources by urban and rural residence largely align with those by socioeconomic status in the next section (Box 2).

BOX 2 **Using Social Marketing Organizations and Social Franchises to Reach Rural and Younger Users in Nepal**

In Nepal, the private sector's role in short-acting method provision is smaller than in neighboring countries, particularly in rural areas. For example, 28% of rural pill users in Nepal rely on private sources compared with 55% in Pakistan, 50% in Bangladesh, and 62% in India. Social marketing and franchising are 2 mechanisms to increase rural short-acting method provision. For example, the Nepal CRS Company, a prominent social marketing organization, uses rural field representatives to increase product availability with pharmacies, which are limited in rural areas. Expanding access to short-acting methods through private outlets such as social marketing organizations and social franchises also benefits adolescents, as data show they disproportionately rely on private sources to obtain their contraception. Read more at https://www.shopsplusproject.org/sources-family-planning-materials.

#### Socioeconomic Status

An efficient market aims to provide all women with access to their choice of contraceptive method and to allocate limited resources where they are needed most. In some markets, this may mean that users with the ability to pay for contraception buy their methods from private sources, enabling the public sector to use its resources to serve individuals without the ability to pay. In this section, we compare contraceptive sources used by women in the wealthiest 20% (quintile) of each country's population with those in the poorest 20% of their country's population. Note that wealth quintiles are a relative measure within each country and are not directly comparable across countries; therefore, while results represent averages across women from the poorest and wealthiest households in each country, women across countries are not all equally wealthy or equally poor.

**FIGURE 4:**
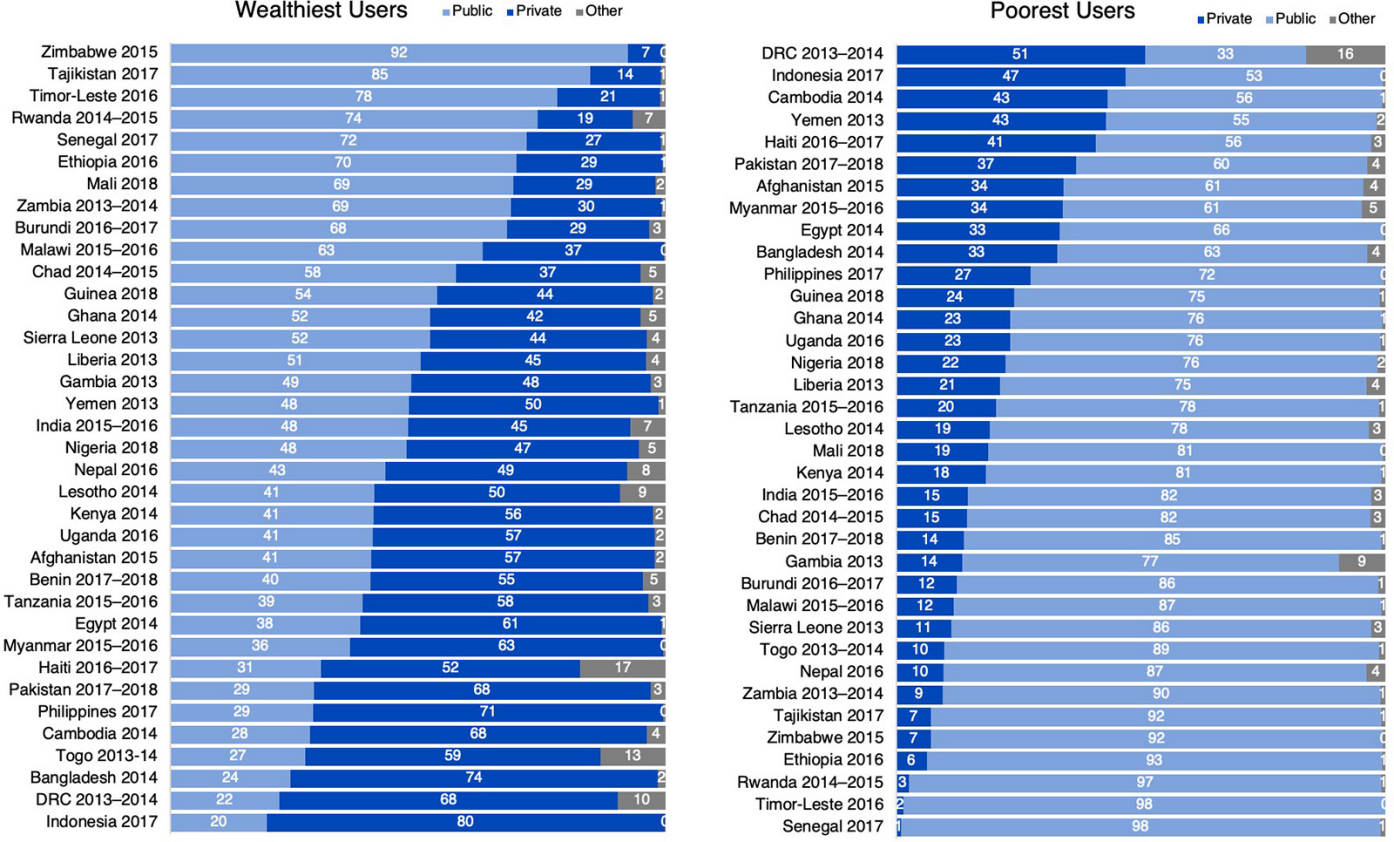
Percentage of Users in the Poorest and Wealthiest Quintiles Obtaining Contraception from Each Sector, by Country Abbreviation: DRC, Democratic Republic of the Congo.

Just over three-fourths (76%) of the poorest users, averaged across countries analyzed, visit public sector sources for their contraception (Table 2). Nearly 1 out of every 4 of the poorest contraceptive users obtains her method from a private source (22%). In some countries, including the DRC, Indonesia, Cambodia, Yemen, Haiti, Pakistan, Afghanistan, and Myanmar, more than one-third of the poorest contraceptive users obtain their method from a private source (Figure 4). Contraceptive sources among the wealthiest women in each country are more evenly split: on average across countries, 46% of the wealthiest women go to public sources and 50% to private sources (Table 2). However, this conceals substantial country variation (Figure 4). The public sector serves more than 70% of the wealthiest contraceptive users in Senegal, Rwanda, Timor-Leste, and Tajikistan, indicating opportunities for more efficient market segmentation. Conversely, the private sector serves more than 70% of the wealthiest users in the Philippines, Bangladesh, and Indonesia (Box 3).

BOX 3 **Increasing Private Sector Provision of Long-Acting Reversible Contraceptives and Permanent Methods in the Philippines Through National Health Insurance**

Although 71% of the wealthiest Filipino contraceptive users rely on private sources, less than one-third rely on private sources for long-acting reversible contraceptives and permanent methods due to limited availability of these methods through private sources. Confronting private sector barriers to provision of long-acting reversible contraceptives and permanent methods would allow wealthier users with the ability to pay for these methods to access them through private facilities, creating opportunities for the government to target its limited resources towards access for the poorest Filipino women. To this end, the Filipino national health insurance, PhilHealth, expanded its accreditation to private providers in 2015. However, receiving training from accredited institutions to obtain PhilHealth reimbursement remains a challenge for private health providers, especially nurses and midwives. Responding to this barrier is key to maximize use of the expanded PhilHealth accreditation and to support a better-segmented and more sustainable family planning market in the Philippines. Read more at https://www.shopsplusproject.org/sources-family-planning-materials.

#### Country Income Status

**FIGURE 5:**
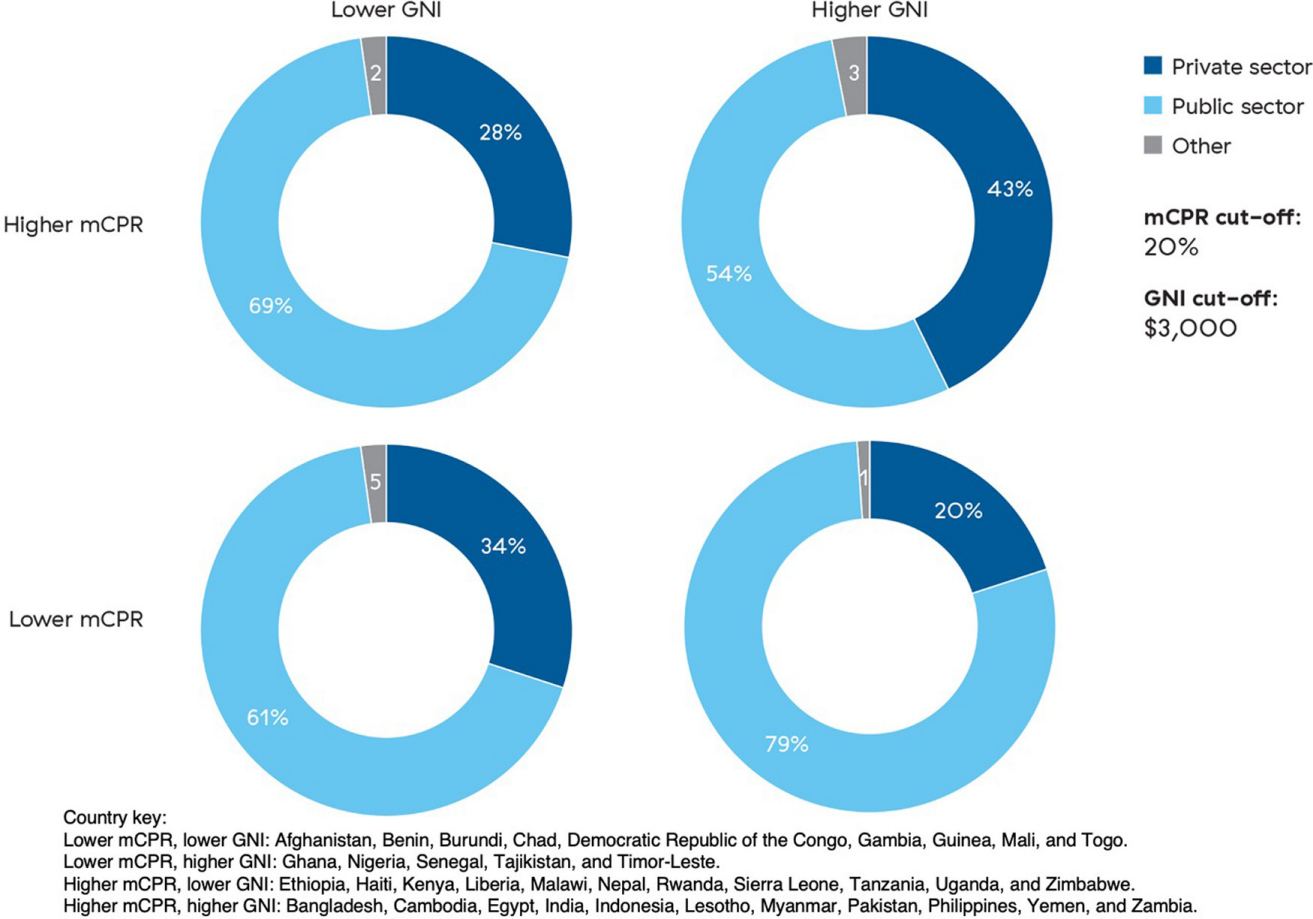
Percentage^a^ of Modern Contraceptive Method Users in Each Country Group That Obtain Contraception From Each Source, by Country Modern Contraceptive Prevalence Rate and Gross National Income per Capita Abbreviations: GNI, gross national income; mCPR, modern contraceptive prevalence rate. ^a^ Percentages may not add up to 100 due to rounding.

Contraceptive source patterns vary greatly by country income status. For example, Indonesia and DRC are 2 countries with the highest percentage of private sector use. However, Indonesia is an upper-middle income country with a high modern contraceptive prevalence rate (mCPR) (41%), while DRC is a low-income country where more than 60% of the population is estimated to live below the poverty line and has an mCPR of 8%. To further explore contraceptive source differences by country income status, we analyzed patterns by GNI per capita (purchasing power parity adjusted)^14^ and mCPR. We split countries into GNI per capita lower and higher than $3,000 and mCPR lower and higher than 20%, creating 4 country groupings with a relatively equal number of countries in each category (Figure 5).

The private sector's role is larger, on average, in countries with lower mCPRs and lower GNI per capita (34%) and in countries with higher mCPRs and higher incomes (43%). The lower-mCPR, lower-income group includes mostly West and Central African countries such as DRC and Guinea but also includes Afghanistan. In these countries, access to contraceptive supplies and services is often limited, and the private sector may be filling gaps left by an underperforming public sector. The private sector in these countries is primarily made up of pharmacies, drug shops, shops, and markets, and many contraceptive supplies are subsidized. Countries in the higher-mCPR and higher-income group represent more mature contraceptive markets, including countries such as Bangladesh, Egypt, India, and the Philippines. A greater share of the private sector is clinical in these countries, often providing LARCs and especially PMs in South Asian countries. In many of these settings, the public and private sectors work together to create more sustainable service delivery. The higher mCPRs in these countries may, in part, reflect successful efforts by both public and private sectors that have expanded contraceptive access and choice, thereby growing the overall contraceptive market.

The private sector's role tends to be larger in countries with lower mCPRs and lower GNI per capita and in countries with higher mCPRs and higher incomes.

The lower-mCPR group with higher per-capita GNIs comprises several West African countries, such as Ghana, Nigeria, and Senegal, as well as Tajikistan and Timor-Leste. These countries may be focusing on a public sector contraceptive response without substantial private sector coordination, which could help to maximize contraceptive access and use. The lower-income, higher-mCPR countries include several East and Southern African countries that have been noted for their strong family planning programs, either historically or recently, including Kenya, Rwanda, and Zimbabwe. In many of these countries, the government has created a public sector infrastructure that is able to meet the contraceptive needs of much of the population.

### Logistic Regression Results

Table 4 displays bivariate and multivariate regression results. Multivariate results adjust for contraceptive method and demographic characteristics (age, marital status, wealth quintile, and urban/rural residence) and confirm the descriptive patterns discussed above. Compared with sterilized women as a reference group, condom use is the largest predictor of private sector use, followed by pill use. Socioeconomic status is also an important predictor of private sector use: compared with the poorest users, the wealthiest users have more than 4 times greater odds of obtaining their method from a private source. Young users, never-married users, and urban users are also more likely to use private sources compared with their older, married, and rural counterparts, respectively.

**TABLE 4.** Unadjusted Odds and Adjusted Odds of Using a Private Sector Source Compared With a Public Sector Source Among All Women Using Modern Contraception^a^

	OR	95% CI	*P* Value	aOR	95% CI	*P* Value
N=365,202						
Contraceptive method (ref: sterilization)						
Pills	7.48	7.14, 7.83	<.001	7.64	7.17, 8.15	<.001
Condoms	15.19	14.20, 16.25	<.001	10.64	9.85, 11.50	<.001
Injectables	2.95	2.80, 3.11	<.001	2.33	2.15, 2.53	<.001
Implants	0.73	0.67, 0.79	<.001	0.61	0.55, 0.68	<.001
IUDs	2.42	2.29, 2.56	<.001	1.58	1.47, 1.70	<.001
Other modern	10.61	7.76, 14.49	<.001	8.73	6.23, 12.22	<.001
Age, years (ref: 40–49)						
15–19	3.30	3.04, 3.59	<.001	2.03	1.81, 2.26	<.001
20–24	2.22	2.12, 2.32	<.001	1.48	1.40, 1.56	<.001
25–29	1.72	1.65, 1.79	<.001	1.36	1.29, 1.43	<.001
30–34	1.51	1.45, 1.57	<.001	1.27	1.22, 1.34	<.001
35–39	1.35	1.30, 1.39	<.001	1.21	1.16, 1.26	<.001
Never married (ref: ever married)	2.90	2.67, 3.15	<.001	1.55	1.37, 1.74	<.001
Wealth quintile (ref: poorest)						
Poorer	1.24	1.18, 1.31	<.001	1.28	1.21, 1.35	<.001
Middle	1.43	1.36, 1.51	<.001	1.53	1.45, 1.63	<.001
Richer	2.02	1.91, 2.13	<.001	2.14	2.01, 2.27	<.001
Richest	3.86	3.64, 4.08	<.001	4.09	3.84, 4.37	<.001
Urban residence (ref: rural)	2.27	2.18, 2.36	<.001	1.42	1.36, 1.48	<.001

Abbreviation: aOR, adjusted odds ratio; CI, confidence interval; IUD, intrauterine device, OR, odds ratio.

a Pooled data from 36 Demographic and Health Surveys. Data are pooled across the most recent survey in all countries analyzed. The adjusted model includes survey fixed effects.

Above we have noted that method use and sociodemographic characteristics are often correlated, particularly with younger and unmarried women more likely to use condoms, which are more frequently sourced from the private sector. The regression results confirm that higher private sector use appears to be independent of method selection for women in these groups. After we control for contraceptive method and other sociodemographic characteristics, adolescents aged 15–19 years still have twice the odds (2.08, *P*<.001) and unmarried women have 1.5 times the odds (1.55, *P*<.001) of obtaining their contraceptives from the private sector, compared with older and married women, respectively. Adjusted regression results also confirm higher odds of private sector use among urban women (1.42, *P*<.001) and women in the wealthiest quintile of their populations (4.09, *P*<.001).

## DISCUSSION

This analysis confirms that both the public and private sectors are important sources of contraception across population segments in nearly all the 36 LMICs examined in this brief. Our finding that the private sector serves, on average, 34% of contraceptive users is in line with earlier global analyses.^6^^,^^7^ This finding is perhaps remarkable given that most global growth in contraceptive prevalence has been attributed to increases in the use of injectables and implants,^1^^,^^15^ which are largely sourced from the public sector. Given this context, it is noteworthy that the private sector has maintained a relatively stable share of the contraceptive market. As contraceptive prevalence and population size both continue to increase, public and private sectors both serve millions of additional users each year.

Both the public and private sectors are important sources of contraception across population segments in nearly all the 36 LMICs examined in this brief.

The private sector is more heterogenous than the public sector and is composed of clinical and nonclinical as well as commercial and nonprofit sources. This diversity of private sector sources is accompanied by distinct factors regarding method choice, availability, and price. These factors have a bearing on users' decisions about sources and methods to use. Our findings demonstrate that clear patterns exist between source and modern contraceptive method. Contraceptive methods are also heterogeneous, with varying effectiveness levels, side effects, and frequency of use and resupply. Condoms and contraceptive pills are the 2 methods most frequently obtained from private sector sources—and specifically from pharmacies or drug shops—and these are also the 2 modern contraceptive methods with the lowest effectiveness levels. While the private sector is primarily a source for short-acting resupply methods, this analysis demonstrates that some injectable, implant, and IUD users rely on private sources—primarily NGOs and FBOs—for these methods.

This analysis demonstrates the importance of the private sector for specific population segments including unmarried women, adolescents, urban residents, and wealthier women. However, the private sector does not only serve these populations. On average across countries analyzed, 1 in every 4 of the poorest contraceptive users and more than 1 of every 4 rural users obtain their method from a private sector source. These data can be used on a country level to inform programs that aim to reach specific population segments. For example, social marketing and social franchising can be used to enhance contraceptive access through the private sector for rural communities, youth, and poorer users through purchasing subsidized methods. Expanding national health insurance coverage to include reimbursement for the private sector is another strategy to expand affordable contraceptive access for current or potential future private sector users. For example, studies show that scaling up national and community-based insurance programs increases access to and voluntary uptake of contraception in countries including Rwanda, Afghanistan, Indonesia, Ghana, and the Philippines.^16^^–^^19^

Quantitative data like those analyzed here can reveal a great deal about where women access contraception. Equally important, although less easy to determine analytically, are the reasons why women access contraception from certain sources or use certain methods. Utilization of the private sector, particularly among poorer women, could indicate a lack of access to public sources in their geographic area.^20^ It may also indicate a preference for the private sector, for reasons of convenience or perceived quality or to ensure their privacy, which is an issue of particular relevance for adolescents and unmarried women.^21^^,^^22^ Women may prefer to access contraception from private sources but may be unable to access all methods there. For example, our analysis showed that nearly one-third of the wealthiest pill users and two-thirds of the wealthiest injectable users rely on public sources. In some countries, this could be related to regulatory barriers that prevent private providers from delivering certain methods, for example, where pharmacists cannot provide pills without prescriptions or face legal restrictions that prevent them from administering injectables at all.^23^ LARCs and PMs, and implants in particular, are frequently more available in public rather than private facilities.^24^ Additionally, even the wealthiest women may be unable to afford the costs of unsubsidized LARCs in the private sector, if they can even be found there. Allowing reimbursement of these methods through national health insurance schemes in the private sector is, again, a key strategy to increase access. These data shed light on barriers to accessing particular methods in particular sectors for particular demographic groups; this evidence can be used to advocate for and design policies and programs that can overcome such barriers.

Quantitative data can reveal where women access contraception, but it is equally important, although less easy, to determine why women use certain sources or certain methods.

Our analysis demonstrates that some groups that have been deemed priorities by multiple countries—particularly adolescents—disproportionately rely on the private sector for their contraception. It is therefore essential that they be able to access all methods within their sector of choice to meet their contraceptive needs to the extent possible and in line with global guidelines.^25^ To increase method choice in the private sector, governments may consider ideas such as strategic purchasing to increase LARC availability and affordability and expansion of LARC subsidies to private providers.^26^ Social marketing and task shifting are 2 additional mechanisms through which to increase private sector method choice.^26^^,^^27^ Governments could also remove legal and regulatory barriers to making short-acting methods available over-the-counter and ensure that as new methods such as subcutaneous depot medroxyprogesterone acetate become available, they can be found through both public and private sector channels. Aggregate data presented in this analysis show potential opportunities for improved resource allocation using a market development approach. However, these patterns must be examined and interpreted at the country and subnational levels with an understanding of multiple factors including service and product availability, mCPR, development status and market maturity, government regulatory policies, and consumer preferences. Reducing policy and regulatory barriers to align country and international standards will maximize the potential impact of both the public and private sectors in family planning and help drive countries' contraceptive markets toward increased sustainability, efficiency, and equity.^28^

### Limitations

We note that the source categorizations used in this study are subject to several limitations and may not perfectly represent all contraceptive sources in every country for several reasons. First, women were asked where they obtained contraception, rather than who provided their service, which could result in some sector misclassifications. For example, in cases in which private providers work in public sector clinics, women likely reported the public clinic rather than the private provider. In addition, women may have reported where they obtained their method, even if it was administered (in the case of injectables), inserted (in the case of implants and IUDs), or prescribed (in the case of pills) by a different provider. A woman may not know the source of her method if it was obtained by her partner, as commonly occurs with condoms. In addition, it is unclear how women reported sources if the contraceptive was delivered to her home, as may be the case with community health worker programs. Finally, an unavoidable degree of uncertainty may be associated with self-reporting, as a woman who attended, for example, an FBO or other NGO clinic may have reported that source as a private clinic rather than naming the clinic as an NGO. Despite these limitations, we believe the opportunities for misclassification in most settings are minor and that estimates based on self-reported sources are generally reliable, in line with previous analyses.

We recognize that all data collected before March 2020 instantly became out of date owing to the coronavirus disease (COVID-19) pandemic. Some data suggest that women switched to self-care methods during the pandemic, including condoms and pills—methods that are primarily distributed through private sector sources.^29^ Therefore, it is possible that since this analysis, the private sector has come to play a larger role in the provision of contraceptives among women who have access, given that contraceptive access has been sharply reduced in many settings.^9^^–^^11^

## CONCLUSION

Despite considerable progress toward FP2020 goals, stark gaps and inequities in contraceptive access and choice remain.^1^ Further, as donor support decreases and LMICs become more self-reliant, much work is needed to realize contraceptive market sustainability. Both the public and private sectors are important sources of modern contraception in nearly every LMIC. Indeed, many women will rely on both public and private sectors as they progress through their reproductive life courses. Harnessing the power and potential of all market actors—government, non-governmental (including faith based, social marketing, and social franchising), and private commercial—is key to accelerating progress toward countries' family planning goals to expand contraceptive access and choice and meet the reproductive needs and preferences of all current and potential future contraceptive users.

## Notes

**Peer Reviewed**
